# Bibliographical

**Published:** 1897-01

**Authors:** 


### Bibliographical.

THE PRINCIPLES OF THEORETICAL CHEMISTRY, with special reference to the Constitution of Chemical Compounds, by IRA REMSEN, Professor of Chemistry in Johns Hopkins University. Fifth edition. Thoroughly revised.

In the preparation of this edition the author, in his preface, remarks: "I have again been tempted to change the book fundamentally and give it a character more in keeping with the recent tendencies in the field of physical and general chemistry, but, taking everything into consideration, I concluded to resist the temptation and remain true to the original character and title of the book. It is a brief treatise on those facts and speculations that have to deal especially with the problem of the constitution of chemical compounds. My object has been to help students to attain clearer ideas in regard to the foundations of chemistry." This work has been popular through its former editions, and has doubtless improved in each issue. In this little volume has been presented in a condensed, and yet very clear manner, the general principles of chemical science, presented so clearly as, in a good degree, at least, to clear away the statement so often made by students that chemistry is a dry and hard study. This volume is an admirable one for the use of the dental and medical student, is it leads on by easy steps from one subject to another and well prepares him for the further prosecution of the work in the more elaborate courses of chemical science. It is a work valuable not only to the student, but is invaluable to the practitioner as a book of reference. It should be found in every dental library. It is obtainable at any book store.

